# Lower triglyceride-cholesterol-body weight index is independently associated with increased in-hospital complication risk: a large multicenter real-world study

**DOI:** 10.1186/s12944-026-02947-w

**Published:** 2026-04-09

**Authors:** Yonghao Li, Yilin Li, Xiaoyi Luan, Liru Chen, Peng Liu, Hongyuan Cui, Mingwei Zhu

**Affiliations:** 1 Department of General Surgery, Department of Hepato-bilio-pancreatic Surgery, Beijing Hospital, National Center of Gerontology, Institute of Geriatric Medicine, Chinese Academy of Medical Sciences, Beijing, 100730 China; 2 Chinese Academy of Medical Sciences, Peking Union Medical College, Beijing, 100730 China; 3 Department of Clinical nutrition, Beijing Hospital, National Center of Gerontology, Institute of Geriatric Medicine, Chinese Academy of Medical Sciences, Beijing, 100730 China

**Keywords:** Triglyceride-Cholesterol-Body weight Index, Hospitalized patients, Complications, Clinical outcomes, Real-world study

## Abstract

**Background:**

Complications significantly impact the prognosis and healthcare burden of hospitalized patients, making early identification of high-risk individuals crucial. While nutritional and metabolic status are influencing factors, existing tools struggle to provide an integrated assessment. The Triglyceride-Cholesterol-Body weight Index (TCBI) is a novel indicator that concurrently reflects both nutritional and metabolic status, yet its value in predicting in-hospital complications remains unclear.

**Methods:**

This observational study leveraged large-scale, multicenter real-world data, enrolling 8,288 eligible hospitalized patients. Demographic information, anthropometric measurements, laboratory results, and clinical outcomes were collected. Due to its skewed distribution, TCBI was analyzed using its natural logarithm-transformed value (TCBI-LN) and categorized into quartiles (Q1-Q4). The primary outcome was the occurrence of complications during hospitalization. Univariate analysis was used to compare inter-group differences. Multivariate logistic regression models were employed to analyze the independent association between TCBI-LN and complication risk. Restricted cubic splines were applied to explore the dose-response relationship. The robustness and generalizability of the association were assessed through subgroup analyses and interaction tests. We further compared five nested logistic regression models incorporating TCBI, its individual components, and existing indices (PNI and TyG) using AUC, NRI, IDI, AIC, and BIC, and performed causal mediation analysis to examine whether complications mediated the associations of TCBI with length of stay (LOS) and hospital cost.

**Results:**

Complications occurred in 403 patients (4.9%). Patients with complications had significantly lower TCBI-LN levels compared to those without (6.83 ± 0.71 vs. 7.10 ± 0.83, *P* < 0.001). Multivariate logistic regression analysis revealed that a higher TCBI-LN remained independently associated with a lower risk of complications even after adjusting for multiple potential confounders, including age, sex, body mass index, disease type, comorbidities, and related prognostic factors (adjusted OR = 0.707, 95% CI: 0.553–0.930, *P* = 0.012). Restricted cubic spline analysis suggested a linear inverse correlation between TCBI-LN and complication risk. Subgroup analyses indicated that the protective association of TCBI-LN was statistically significant in males, patients aged < 65 years, those with a body mass index < 18.5 or ≥ 24 kg/m², and malnourished patients. No significant interactions were observed across all subgroups (P for interaction > 0.05). A risk stratification cutoff was determined based on the Youden index. The complication rate was significantly higher in the high-risk group (6.3%) compared to the intermediate- (5.1%) and low-risk groups (2.9%). In model comparison, adding TCBI-LN to a clinical model significantly improved AUC, NRI and IDI, and the model combining TCBI-LN with PNI and TyG provided the best overall performance. Mediation analysis indicated that TCBI-LN shortened LOS predominantly through reducing in-hospital complications and partially attenuated its direct cost-increasing effect.

**Conclusion:**

In a large-scale cohort study of hospitalized patients, lower TCBI-LN levels were independently associated with a higher risk of in-hospital complications, and this association was generalizable across different patient subgroups. As a composite index easily derived from routine laboratory tests, TCBI may serve as a practical tool for early identification of patients at high risk for in-hospital complications and ultimately improve clinical outcomes.

**Supplementary Information:**

The online version contains supplementary material available at 10.1186/s12944-026-02947-w.

## Introduction

The occurrence of complications during hospitalization is a critical factor affecting clinical outcomes, prolonging hospital stays, and increasing healthcare resource utilization. According to a systematic review, the median incidence rate of in-hospital complications is 9.2% (IQR 4.6–12.4%) [1]. These complications not only directly lead to increased patient mortality and impede recovery but are also closely associated with a significant rise in hospitalization cost (HC) and average length of stay (LOS) [2–5]. Therefore, the early and accurate identification of patient populations at high risk for complications is of paramount clinical importance for implementing targeted preventive strategies, optimizing the allocation of clinical resources, and ultimately improving patient prognosis. The development of complications is a complex process involving multiple contributing factors, among which a patient’s baseline nutritional status and metabolic state are considered two key modifiable intrinsic risk factors [6, 7]. Malnutrition has been clearly linked to adverse clinical outcomes such as infection and poor wound healing [8]. Current clinical tools commonly used for nutritional screening and assessment, such as the Nutrition Risk Screening 2002 (NRS 2002) and the Global Leadership Initiative on Malnutrition (GLIM) criteria, primarily focus on evaluating phenotypic indicators like food intake, weight change, and muscle mass [9]. However, these tools offer relatively limited integrated assessment of a patient’s metabolic state. On the other hand, metabolic abnormalities, particularly dyslipidemia and insulin resistance, which are associated with chronic low-grade inflammation, endothelial dysfunction, and a higher risk of cardiovascular events, have also been identified as significant predictors of in-hospital complications [10, 11]. For instance, the Triglyceride-Glucose Index (TyG Index), a simple surrogate marker of insulin resistance, has shown some prognostic predictive value [12]. Nevertheless, traditional single-dimensional indicators often struggle to fully capture the intricate interplay between nutrition and metabolism. For example, an obese patient might simultaneously present with overnutrition and atherogenic dyslipidemia, whereas a frail elderly patient might face the dual challenge of malnutrition and metabolic disease. There is a pressing clinical need for composite biomarkers capable of simultaneously reflecting the body’s nutritional reserves and metabolic homeostasis.

In recent years, the Triglyceride-Cholesterol-Body weight Index (TCBI) has been proposed as a novel composite index. It is calculated using the formula: TCBI = Triglyceride (TG) (mg/dL) × Total Cholesterol (TC) (mg/dL) × Body Weight (BW) (kg) / 1000. This index innovatively integrates BW, which reflects energy reserves and body composition, with TG and TC, which are core circulating markers of lipid metabolism. Preliminary studies suggest that TCBI may be associated with the risk of coronary artery disease [13]. It potentially offers a more comprehensive picture of the pathophysiological state than single indicators by encompassing both nutritional and metabolic dimensions. Furthermore, all its component variables are routinely measured upon hospital admission, making it easily obtainable, cost-effective, and highly feasible for clinical use. However, the value of TCBI for predicting in-hospital complications—a composite clinical outcome—in a broader, more heterogeneous population of hospitalized patients remains unclear, lacking large-scale prospective evidence.

In summary, this study aims to systematically investigate the predictive value of TCBI for the occurrence of complications in hospitalized patients, based on a multicenter real-world study. The goal is to provide clinicians with a novel, comprehensive, and practical biomarker to aid in the early identification of high-risk patients and to lay the groundwork for further exploration of its interventional value.

## Methods

### Study population

This study was conducted using data from two large, multicenter, prospective cohorts, involving a total of 17,306 patients. The first study was conducted from March to May 2012 and consecutively enrolled hospitalized patients from 14 tertiary hospitals across mainland China. Its protocol was approved by the Ethics Committee of Beijing Hospital (Registration No.: LLKYPJ2012002A). The second study was a multicenter investigation carried out from June to September 2014 across 34 tertiary hospitals in mainland China, during which eligible patients were consecutively enrolled. This study protocol was approved by the Ethics Committee of Beijing Hospital (Registration No.: 2014BJYYEC-022-02) and registered with the Chinese Clinical Trial Registry (Registration No.: ChiCTR-EPC-14005253).

**Fig. 1:**
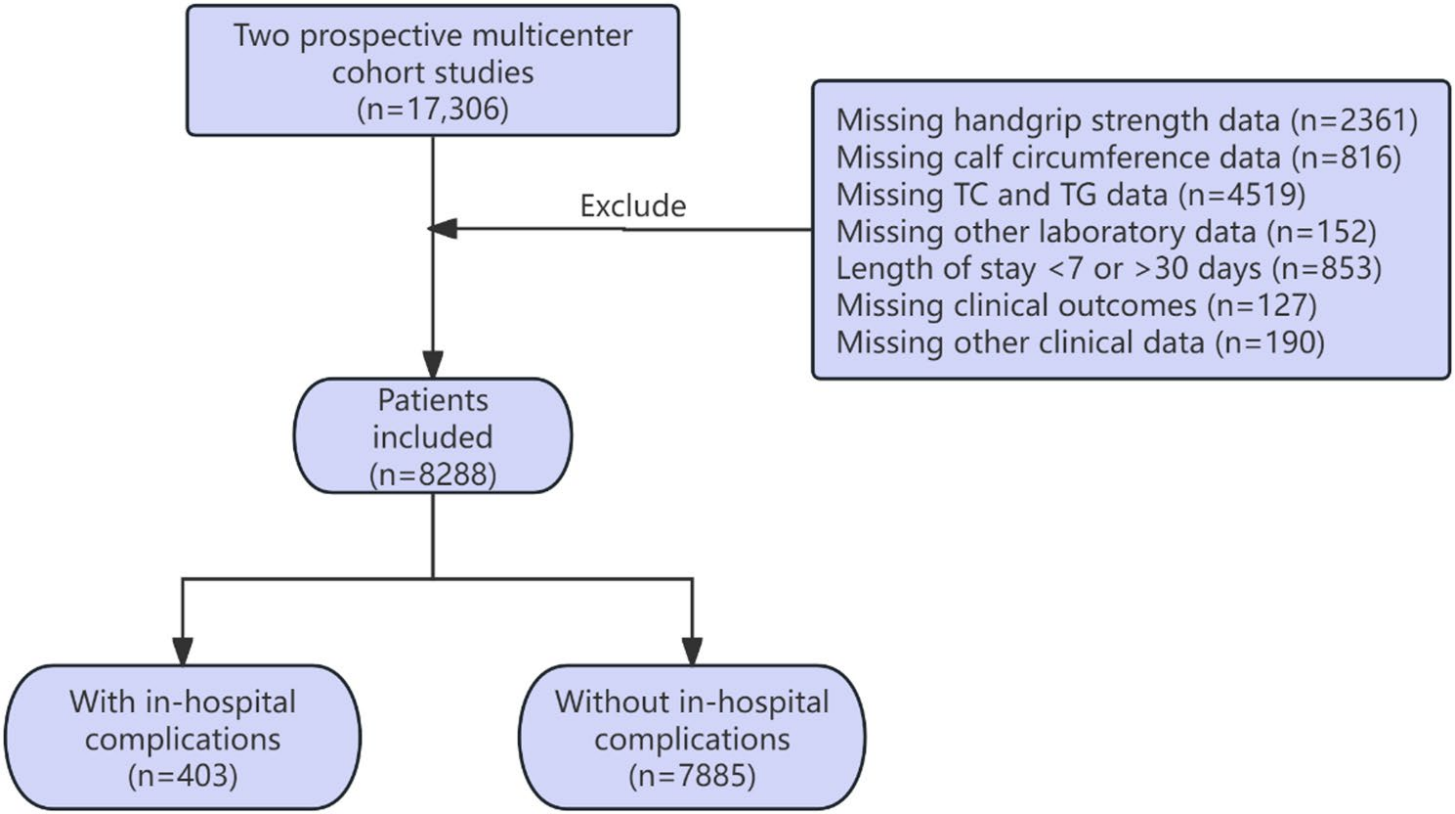
Flowchart of patient selection for the present study

Inclusion criteria were: (1) conscious and alert, (2) not undergoing emergency surgery, and (3) a hospital stay between 7 and 30 days. Exclusion criteria included: (1) admission through the emergency department, (2) refusal to participate or provide informed consent, (3) missing clinical data during hospitalization, and (4) a hospital stay of less than 7 days or more than 30 days. The patient screening flowchart is presented in Fig. 1.

### Data collection

A standardized study protocol was employed. All laboratory and anthropometric measurements were completed within 24 h of admission. Collected data included:

(1) Demographic parameters: sex, age, marital status, and education level. (2) Clinical parameters: reason for hospitalization, medical history, weight loss, and food intake. (3) Anthropometric parameters: height, BW, calf circumference (CC), and handgrip strength (HGS), all measured using standard methods. All research personnel received standardized training on obtaining anthropometric data. For CC measurement, participants were seated on a chair with knees bent at 90° and feet flat on the floor; a non-stretchable tape measure was used to measure the thickest part of the calf. HGS was measured using an electronic dynamometer (EH101, Xiangshan, Guangdong, China). Participants were seated with shoulders adducted and elbows flexed at 90°. Three measurements were taken for each hand, and the maximum value was recorded to the nearest 0.1 kg. (4) Laboratory parameters: complete blood count and blood biochemistry, including total protein (TP), albumin, TG, and TC. The platelet-to-lymphocyte ratio (PLR) was calculated from complete blood count results. The prognostic nutritional index (PNI) was calculated as: PNI = 10 × serum albumin (g/dL) + 5 × total lymphocyte count (×10⁹/L) [14]. The TyG index was calculated as: TyG = Ln [TG (mg/dL) × fasting plasma glucose (mg/dL) / 2] [12]. Following established methodology, TCBI was calculated as: TCBI = TG (mg/dL) × TC (mg/dL) × BW (kg) / 1000 [15].

All patients were assessed using the NRS 2002 tool within 24 h of admission [16]. Implementation of the GLIM criteria [9] involved a two-step process: ① screening for nutritional risk (NRS 2002 score ≥ 3); ② diagnosing malnutrition based on phenotypic and etiologic criteria endorsed by the GLIM committee. Phenotypic criteria included: (1) Weight loss: unintentional weight loss > 5% within the past 6 months, or > 10% beyond 6 months. (2) Low body mass index (BMI): for individuals < 70 years, BMI < 18.5 kg/m²; for those ≥ 70 years, BMI < 20 kg/m², per GLIM committee suggestions for Asian populations [9]. (3) Reduced muscle mass: Due to the lack of body composition data in the dataset, two proxy criteria were used. Criterion 1: reduced CC (male < 34 cm, female < 33 cm). Criterion 2: reduced HGS (age ≥ 65: male < 28 kg, female < 18 kg; age < 65: male < 34 kg, female < 20 kg). Reduced muscle mass was considered positive if both criteria were met, aligning with previous studies and the 2025 Asian Working Group for Sarcopenia (AWGS) consensus update [17]. Etiologic criteria included: (1) Reduced food intake or assimilation: energy intake ≤ 50% of requirement for > 1 week, or any reduction for > 2 weeks, or the presence of chronic gastrointestinal conditions affecting intake/absorption. (2) Disease burden/inflammation: presence of acute or chronic disease-related inflammation, assessed via patient history. In this study, low serum albumin (< 35 g/L) was used as an inflammation indicator [18, 19]. To assess disease severity, we created a composite disease severity score by summing the following indicators: ICU admission (1 point), anemia (1 point), albumin < 35 g/L (1 point), and NRS disease severity score ≥ 2 (1 point), with scores ranging from 0 to 4.

### Adverse clinical outcomes

The adverse clinical outcomes in this study included the occurrence of various complications during hospitalization, LOS, and HC. Complications were defined according to the Clavien-Dindo classification system, with grades II-V included in the analysis. These were further categorized as: (1) infection-related complications (surgical site infection, pneumonia, urinary tract infection, bloodstream infection, and catheter-related infection); and (2) non-infection-related complications (anastomotic leakage, cardiovascular events including myocardial infarction and arrhythmias, respiratory failure, acute kidney injury, electrolyte disturbances requiring intervention, anemia requiring blood transfusion, thromboembolic events, and wound dehiscence). Prior to study initiation, all participating physicians received standardized training on complication identification and recording. Complications were prospectively identified and documented by the treating clinical team during daily ward rounds. For ambiguous cases, adjudication was performed through discussion with senior attending physicians from the relevant departments. The central coordinating team at Beijing Hospital reviewed all submitted data for consistency and completeness.

### Statistical analysis

Data were analyzed using R software (Version 4.4.3). Continuous variables are presented as mean ± standard deviation (normally distributed) or median with interquartile range (skewed distribution). Categorical variables are presented as frequencies and percentages. Group comparisons for continuous variables used one-way ANOVA or the Kruskal-Wallis H test, while categorical variables were compared using the chi-square test or Kruskal-Wallis H test. Due to its skewed distribution, TCBI was natural log-transformed (TCBI-LN) to approximate normality for analysis. Pearson’s or Spearman’s correlation coefficients were used to assess correlations between baseline parameters and TCBI. Univariate analysis was performed to examine the associations between TCBI, other clinical characteristics, and complications. Subsequently, multivariable logistic regression analysis was used to evaluate the relationship between TCBI-LN and the occurrence of in-hospital complications, with results expressed as odds ratios (OR) per unit increase and their corresponding 95% confidence intervals (CI). The selection of covariates in this study was based on a two-step approach. We integrated findings from univariate analyses with results from previous studies on complications and nutritional indicators in hospitalized patients to identify potentially relevant confounders. Key confounders included demographic characteristics, anthropometric measurements, nutritional indicators, disease characteristics, comorbidities, and other prognostic markers. Subsequently, we constructed directed acyclic graphs (DAGs) incorporating these potential confounders to identify the necessary adjustment variables. Based on these results, we constructed a crude model and sequentially adjusted models as follows: Model 1 (crude model); Model 2 adjusted for sex, age, marital status, educational level, and ethnicity; Model 3 further adjusted for department type, disease category, hypertension, diabetes, coronary heart disease (CHD), and cerebral infarction. Model 3 represented the minimally sufficient adjustment set derived from the DAGs. Building upon this, Model 4 additionally adjusted for patient malnutrition, CC, HGS, and BMI; Model 5 further adjusted for predictive indices including PNI, TyG, and PLR; and Model 6 additionally adjusted for disease severity grade. Multicollinearity was checked using the variance inflation factor (VIF). Furthermore, restricted cubic splines (RCS) were applied to visualize potential nonlinear relationships between TCBI-LN and complication occurrence.

Patients were categorized into high-, intermediate-, and low-risk groups based on a cutoff value determined by maximizing the Youden index. Complication rates were compared among these groups. Finally, stratified logistic regression analyses were conducted across subgroups defined by sex, age, BMI (< 18.5 or ≥ 24.0 kg/m² vs. 18.5–24.0 kg/m²), nutritional status, department type, and history of hypertension, CHD, diabetes, or cerebral infarction to explore the relationship between TCBI-LN and complications. Interaction effects were tested using the likelihood ratio test. For sensitivity analysis, we employed multilevel mixed-effects logistic regression models with hospital as a random intercept, as well as generalized estimating equations (GEE) with an exchangeable correlation structure and cluster-robust sandwich standard errors, to evaluate potential clustering effects at the hospital level. To identify differences across LOS and according to whether patients experienced ICU admission during hospitalization, we performed subgroup analyses using binary logistic regression. Additionally, to assess the incremental predictive value of TCBI-LN beyond traditional clinical variables and previously established nutritional/metabolic indicators, we constructed five nested logistic regression models (Models A–E), incorporating individual components of TCBI, TCBI-LN, PNI, TyG, and other indices. The discriminative and reclassification performance of each model was compared using the area under the receiver operating characteristic curve (AUC) with DeLong’s test, likelihood ratio test (LRT), Akaike information criterion (AIC), Bayesian information criterion (BIC), net reclassification improvement (NRI), and integrated discrimination improvement (IDI). Finally, mediation analysis was performed to explore the relationship between TCBI-LN and both LOS and HC. All tests were two-sided, and a P-value < 0.05 was considered statistically significant.

## Results

### Clinical characteristics according to TCBI quartiles

**Fig. 2:**
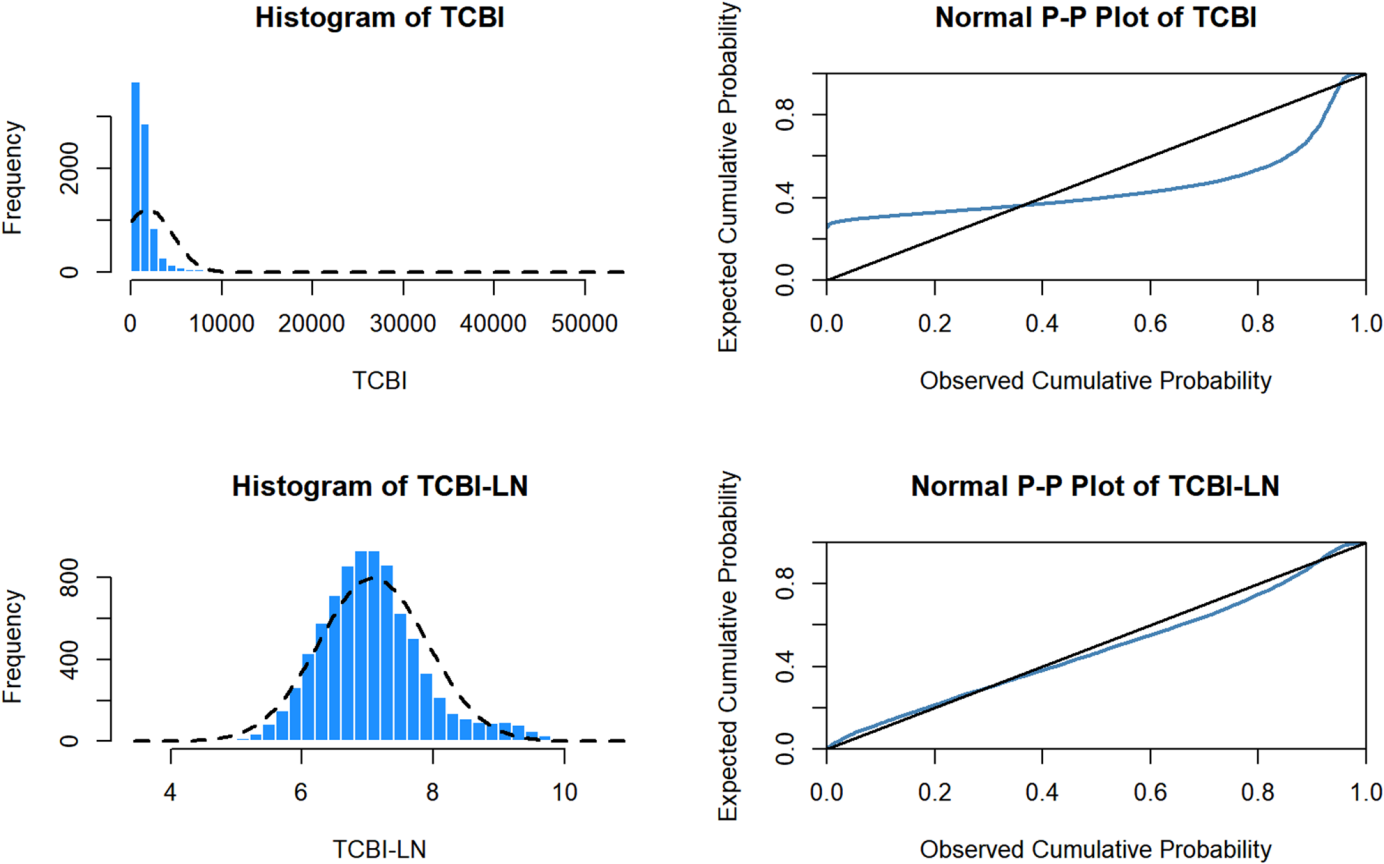
Distribution characteristics of TCBI and TCBI-LN. **a** Histogram of TCBI. **b** Normal P-P plot of TCBI. **c** Histogram of TCBI-LN. **d** Normal P-P plot of TCBI-LN

A total of 8,288 hospitalized patients were included in the final analysis. As the original TCBI variable exhibited a skewed distribution, it was natural log-transformed, resulting in an approximately normally distributed variable (TCBI-LN) (Fig. 2). Based on TCBI-LN quartile cutoff values, patients were divided into four groups (Q1–Q4), each containing 2,072 patients. The baseline characteristics of these groups are presented in Table 1.

**Table 1:** Baseline clinical characteristics of the study population stratified by TCBI-LN quartiles

Characteristics	Overall (*n* = 8288)	Quartile 1 (*n* = 2072)	Quartile 2 (*n* = 2072)	Quartile 3 (*n* = 2072)	Quartile 4 (*n* = 2072)	*P* value	Statistic
Sex, n (%)						0.001	15.705
Male	5096 (61.5%)	1340 (64.7%)	1287 (62.1%)	1240 (59.8%)	1229 (59.3%)		
Female	3192 (38.5%)	732 (35.3%)	785 (37.9%)	832 (40.2%)	843 (40.7%)		
Age(year), mean ± sd	67.47 ± 13.81	68.93 ± 14.95	69.65 ± 12.77	68.14 ± 12.43	63.15 ± 14.01	<0.001	94.213
Marital status, n (%)						0.039	8.349
Married	7977 (96.2%)	1982 (95.7%)	2012 (97.1%)	2000 (96.5%)	1983 (95.7%)		
Single or divorced	311 (3.8%)	90 (4.3%)	60 (2.9%)	72 (3.5%)	89 (4.3%)		
Weight(kg), mean ± sd	63.97 ± 11.61	57.63 ± 10.47	62.69 ± 10.65	66.54 ± 10.68	69.03 ± 11.32	<0.001	439.608
Ethnicity, n (%)						0.022	9.589
Han Chinese	7984 (96.3%)	1980 (95.6%)	1986 (95.8%)	2007 (96.9%)	2011 (97.1%)		
Others	304 (3.7%)	92 (4.4%)	86 (4.2%)	65 (3.1%)	61 (2.9%)		
Education Level, n (%)						<0.001	69.878
Bachelor’s degree or above	2622 (31.6%)	664 (32%)	675 (32.6%)	655 (31.6%)	628 (30.3%)		
Junior high school or below	3702 (44.7%)	869 (41.9%)	905 (43.7%)	894 (43.1%)	1034 (49.9%)		
Senior high school	1011 (12.2%)	259 (12.5%)	217 (10.5%)	280 (13.5%)	255 (12.3%)		
Unknown	953 (11.5%)	280 (13.5%)	275 (13.3%)	243 (11.7%)	155 (7.5%)		
Payment Method, n (%)						0.003	24.683
Public funding (government)	931 (11.2%)	230 (11.1%)	240 (11.6%)	259 (12.5%)	202 (9.7%)		
Basic medical insurance	5405 (65.2%)	1308 (63.1%)	1360 (65.6%)	1374 (66.3%)	1363 (65.8%)		
Out-of-pocket	1601 (19.3%)	452 (21.8%)	389 (18.8%)	356 (17.2%)	404 (19.5%)		
Others	351 (4.2%)	82 (4.0%)	83 (4.0%)	83 (4.0%)	103 (5.0%)		
Hospital Department, n (%)						0.002	15.366
Medical department	5218 (63.0%)	1344 (64.9%)	1263 (61.0%)	1260 (60.8%)	1351 (65.2%)		
Surgical department	3070 (37.0%)	728 (35.1%)	809 (39.0%)	812 (39.2%)	721 (34.8%)		
Primary Diagnosis, n (%)						<0.001	495.933
Endocrine and Rheumatic Immune Diseases	278 (3.4%)	67 (3.2%)	66 (3.2%)	66 (3.2%)	79 (3.8%)		
Neurological and Psychiatric Diseases	1390 (16.8%)	222 (10.7%)	268 (12.9%)	298 (14.4%)	602 (29.1%)		
Bone and Joint Diseases	532 (6.4%)	89 (4.3%)	121 (5.8%)	147 (7.1%)	175 (8.4%)		
Benign Digestive System Diseases	1406 (17%)	425 (20.5%)	342 (16.5%)	336 (16.2%)	303 (14.6%)		
Benign genitourinary diseases	150 (1.8%)	38 (1.8%)	38 (1.8%)	41 (2%)	33 (1.6%)		
Benign Respiratory System Diseases	834 (10.1%)	333 (16.1%)	232 (11.2%)	171 (8.3%)	98 (4.7%)		
Cardiovascular Diseases	1217 (14.7%)	285 (13.8%)	330 (15.9%)	339 (16.4%)	263 (12.7%)		
Malignant Tumors	2291 (27.6%)	566 (27.3%)	624 (30.1%)	628 (30.3%)	473 (22.8%)		
Others	190 (2.3%)	47 (2.3%)	51 (2.5%)	46 (2.2%)	46 (2.2%)		
History of Diabetes, n (%)						<0.001	18.917
Yes	1064 (12.8%)	216 (10.4%)	272 (13.1%)	309 (14.9%)	267 (12.9%)		
No	7224 (87.2%)	1856 (89.6%)	1800 (86.9%)	1763 (85.1%)	1805 (87.1%)		
History of Hypertension, n (%)						<0.001	39.995
Yes	2595 (31.3%)	556 (26.8%)	656 (31.7%)	744 (35.9%)	639 (30.8%)		
No	5693 (68.7%)	1516 (73.2%)	1416 (68.3%)	1328 (64.1%)	1433 (69.2%)		
History of Coronary Heart Disease, n (%)						<0.001	31.473
Yes	1214 (14.6%)	311 (15.0%)	331 (16.0%)	344 (16.6%)	228 (11.0%)		
No	7074 (85.4%)	1761 (85.0%)	1741 (84.0%)	1728 (83.4%)	1844 (89.0%)		
History of Cerebral Infarction, n (%)						0.175	4.962
Yes	744 (9.0%)	164 (7.9%)	200 (9.7%)	198 (9.6%)	182 (8.8%)		
No	7544 (91.0%)	1908 (92.1%)	1872 (90.3%)	1874 (90.4%)	1890 (91.2%)		
Calf Circumference(cm), mean ± sd	33.13 ± 5.19	31.66 ± 3.69	33.12 ± 7.88	33.86 ± 3.49	33.88 ± 4.08	<0.001	162.710
Handgrip Strength(kg), median (IQR)	23 (13, 32)	20 (12.48, 29)	22 (13, 31.50)	24 (14, 34)	25 (15.98, 35.13)	<0.001	114.481
BMI(kg/m^2^), mean ± sd	23.47 ± 3.58	21.34 ± 3.30	23.06 ± 3.20	24.41 ± 3.25	25.04 ± 3.40	<0.001	514.462
NRS 2002, n (%)						<0.001	608.607
<3	5071 (61.2%)	887 (42.8%)	1213 (58.5%)	1372 (66.2%)	1599 (77.2%)		
3–5	2492 (30.1%)	846 (40.8%)	655 (31.6%)	592 (28.6%)	399 (19.3%)		
≥ 5	725 (8.7%)	339 (16.4%)	204 (9.8%)	108 (5.2%)	74 (3.6%)		
Nutritional status (GLIM), n (%)						<0.001	409.619
Malnutrition	2397 (28.9%)	925 (44.6%)	635 (30.6%)	448 (21.6%)	389 (18.8%)		
No malnutrition	5891 (71.1%)	1147 (55.4%)	1437 (69.4%)	1624 (78.4%)	1683 (81.2%)		
WBC(×10⁹/L), median (IQR)	6.1 (4.9, 7.6)	5.7 (4.45, 7.5)	5.9 (4.8, 7.24)	6.1 (5, 7.4)	6.6 (5.26, 8.28)	<0.001	199.754
LYM(×10⁹/L), median (IQR)	1.6 (1.14, 2.11)	1.3 (0.93, 1.8)	1.5 (1.1, 2)	1.65 (1.23, 2.1)	1.99 (1.4, 2.6)	<0.001	692.229
Hb(g/L), median (IQR)	127 (115, 139)	121 (107, 133)	127 (114, 137)	130 (119, 141)	131 (121, 143)	<0.001	432.609
PLT(×10⁹/L), median (IQR)	202 (178, 230)	196 (169, 229)	200 (178, 227)	203 (181, 229)	207 (184, 234)	<0.001	57.143
PLR, median (IQR)	127.25 (95.22, 176.44)	148.14 (109.27, 212.29)	131.85 (100.91, 181.15)	123.55 (97.22, 168.3)	108.55 (78.48, 147.92)	<0.001	446.978
GLU(mg/dl), median (IQR)	103.09 (92.70,115.38)	100.8 (90, 110.7)	101.67 (91.8, 111.3)	102.96 (93.39, 113.65)	108.91 (96.64, 127.59)	<0.001	264.967
TyG, median (IQR)	8.64 (8.27, 9.10)	8.08 (7.85, 8.32)	8.45 (8.26, 8.66)	8.76 (8.58, 8.96)	9.40 (9.07, 10.10)	<0.001	5029.213
ALT(U/L), median (IQR)	17.34 (12, 26)	15.7 (11, 23)	16.59 (12, 23.52)	18 (13, 24.41)	20 (14, 31)	<0.001	265.738
TBIL(µmol/L), median (IQR)	11.4 (8.2, 15.1)	11.5 (8.2, 15.9)	11.41 (8.5, 15.2)	11.6 (8.50, 15)	11 (7.8, 14.6)	0.001	17.505
BUN(mmol/L), median (IQR)	5.5(4.4, 6.7)	5.47 (4.28, 6.83)	5.48 (4.34, 6.7)	5.5 (4.54, 6.6)	5.5 (4.49, 6.7)	0.619	1.783
Cr(µmol/L), median (IQR)	71 (59, 84)	70 (58.95, 83)	72 (60.58, 85)	73 (61, 85)	69 (55.88, 83)	<0.001	49.497
TG(mg/dl), median (IQR)	109.83 (77.94, 161.20)	63.77 (53.14, 77.06)	92.113 (78.83, 108.06)	123.11 (107.96, 143.48)	219.21 (164.74, 412.96)	<0.001	5573.619
TC(mg/dl), median (IQR)	162.89 (135.35, 191.42)	133.02 (110.6, 154.68)	156.61 (135.34, 177.88)	172.08 (150.81, 196.15)	194.9 (170.15, 225.06)	<0.001	2296.147
ALB(g/L), median (IQR)	39 (35.7, 42)	36.8 (33.42, 40)	38.3 (35.24, 41)	39.24 (36.8, 42)	41 (37.8, 44)	<0.001	785.544
PNI, median (IQR)	47 (42.6, 51.75)	43.65 (39.4, 48.15)	46.1 (42.3, 50.3)	47.75 (44, 51.61)	50.905 (46, 56.21)	<0.001	1049.670
TP(g/L), median (IQR)	65.9 (61.2, 70.2)	63.9 (59.6, 68.61)	65 (60.8, 69)	66 (62, 70)	68.56 (64, 72.7)	<0.001	476.956
Overall complications, n (%)						<0.001	29.512
Yes	403 (4.9%)	133 (6.4%)	106 (5.1%)	105 (5.1%)	59 (2.8%)		
No	7885 (95.1%)	1939 (93.6%)	1966 (94.9%)	1967 (94.9%)	2013 (97.2%)		
Infection-related complications, n (%)						<0.001	21.861
Yes	331 (4.0%)	106 (5.1%)	89 (4.3%)	87 (4.2%)	49 (2.4%)		
No	7957 (96%)	1966 (94.9%)	1983 (95.7%)	1985 (95.8%)	2023 (97.6%)		
Non-infection-related Complications, n (%)						<0.001	19.212
Yes	391 (4.7%)	127 (6.1%)	103 (5%)	94 (4.5%)	67 (3.2%)		
No	7897 (95.3%)	1945 (93.9%)	1969 (95.0%)	1978 (95.5%)	2005 (96.8%)		
Total length of hospital stay(days), mean ± sd	13.53 ± 5.92	13.91 ± 6.28	13.71 ± 6.15	13.28 ± 5.85	13.21 ± 5.31	<0.001	6.811
ICU admission, n (%)						<0.001	253.820
Yes	411 (5.0%)	62 (3.0%)	56 (2.7%)	54 (2.6%)	239 (11.5%)		
No	7877 (95.0%)	2010 (97.0%)	2016 (97.3%)	2018 (97.4%)	1833 (88.5%)		
Total healthcare costs(CNY), median (IQR)	19792.8 (10467.6, 45341.3)	18013.8 (10548.1, 37921.2)	18636.0 (10133.0, 44124.9)	19876.9 (10394.0, 49713.5)	23451.7 (11050.0, 48715.0]	<0.001	10.299
Nutritional Support Modalities During Hospitalization, n (%)						<0.001	43.274
No	6097 (73.6%)	1469 (70.9%)	1559 (75.2%)	1540 (74.3%)	1529 (73.8%)		
PN	1168 (14.1%)	355 (17.1%)	267 (12.9%)	250 (12.1%)	296 (14.3%)		
EN	483 (5.8%)	105 (5.1%)	126 (6.1%)	153 (7.4%)	99 (4.8%)		
Both	540 (6.5%)	143 (6.9%)	120 (5.8%)	129 (6.2%)	148 (7.1%)		
In-hospital mortality, n (%)						0.571	2.007
Yes	28 (0.3%)	10 (0.5%)	7 (0.3%)	6 (0.3%)	5 (0.2%)		
No	8260 (99.7%)	2062 (99.5%)	2065 (99.7%)	2066 (99.7%)	2067 (99.8%)		

**Fig. 3:**
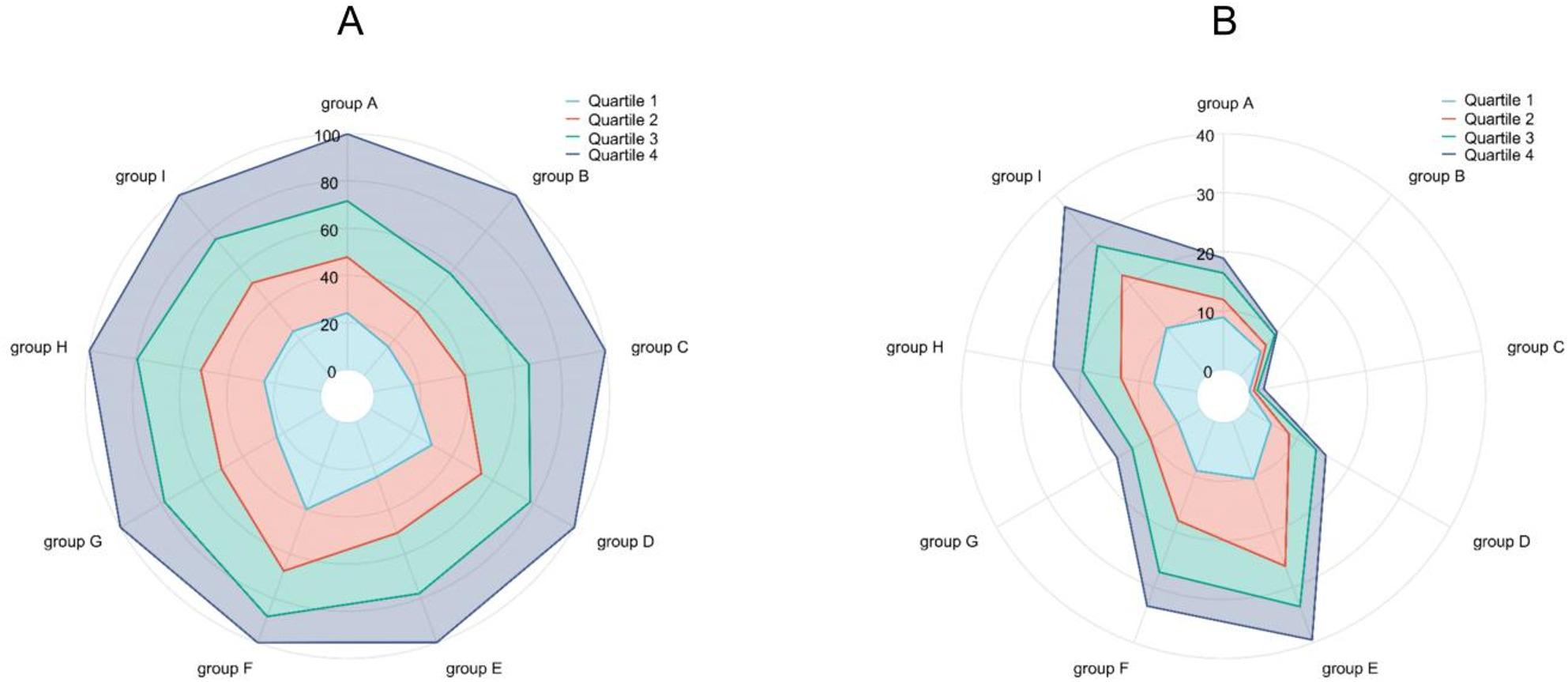
Distribution of TCBI-LN across different disease types: (**A**) Percentage of patients in each TCBI-LN quartile (%); (**B**) Percentage of complication rates in each TCBI-LN quartile (%). Note: group A: Endocrine and rheumatic immune diseases; group B: Neurological and psychiatric diseases; group C: Bone and joint diseases; group D: Benign digestive system diseases; group E: Benign genitourinary diseases; group F: Benign respiratory system diseases; group G: Cardiovascular diseases; group H: Malignant tumors; group I: Other diseases.

With increasing TCBI-LN levels, patients’ age gradually decreased, and the proportion of males was relatively higher in the high TCBI-LN group. Compared with the lowest quartile group, patients in the highest quartile group showed significantly higher BMI, BW, and CC. Furthermore, nutrition-related indicators, such as serum albumin and TP, exhibited an increasing trend, while the proportion of patients with nutritional risk/malnutrition (NRS 2002 score ≥ 3, GLIM diagnosis positive) decreased significantly. As the TCBI-LN quartile increased, lipid-related parameters and the TyG index increased, whereas inflammatory markers such as the PLR decreased. In addition, clinical outcomes including the complication rate and ICU admission rate also differed significantly among the quartile groups (*p* < 0.001). The distribution of TCBI-LN also varied significantly across different disease types (*p* < 0.001). For instance, patients with neurological diseases had a relatively higher proportion in the high TCBI-LN interval (Q4) (46.3%), while patients with benign respiratory diseases were more concentrated in the low TCBI-LN interval (Q1) (39.9%). Detailed distributions of TCBI-LN and complication rates across different diseases are illustrated in Fig. 3.

### Correlation analysis between TCBI-LN and clinical characteristics

**Fig. 4:**
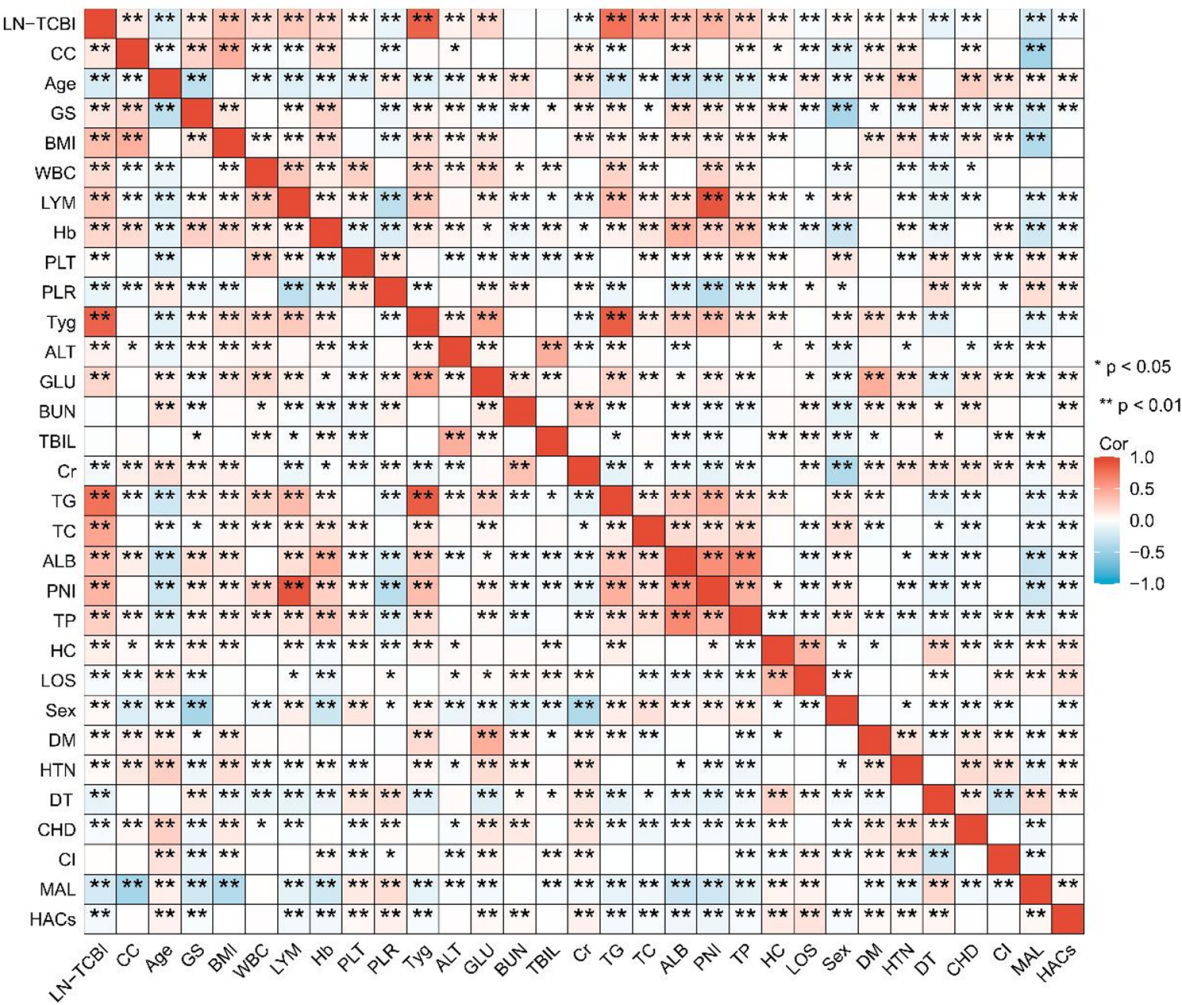
Correlation analysis between TCBI-LN and clinical parameters. Note: CC: Calf Circumference; GS: Grip Strength; WBC: White Blood Cell; LYM: Lymphocyte; PLT: Platelet; ALT: Alanine Aminotransferase; GLU: Glucose; TBIL: Total Bilirubin; Cr: Creatinine; TP: Total Protein; HC: Hospitalization Cost; LOS: Length of Stay; DM: Diabetes; HTN: Hypertension; CHD: Coronary Heart Disease; CI: Cerebral Infarction; MAL: Infection-related Complications; HACs: Non-infection-related Complications; DT: Disease Type

Correlation analysis (Fig. 4) revealed that TCBI-LN exhibited moderately positive correlations with nutrition-related indicators, including BMI, CC, HGS, albumin, TP, and the PNI. The correlation coefficients were concentrated in the moderate range, and all were statistically significant (*P* < 0.01). In contrast, TCBI-LN was negatively correlated with age and inflammatory markers such as the PLR, while showing positive correlations with lipid metabolism markers, including TG, TC, and the TyG index. This pattern suggests that TCBI-LN reflects a composite picture of a patient’s nutritional and metabolic status. Regarding prognosis-related indicators, TCBI-LN showed a weak negative correlation with HC, indicating that lower TCBI-LN was generally associated with higher costs. Furthermore, the correlation coefficients between TCBI-LN and both infection-related and non-infection-related complications were negative and statistically significant.

### Predictive value of TCBI-LN for in-hospital complications

As shown in Table 2, the univariate analysis revealed that patients who developed in-hospital complications had significantly lower TCBI-LN levels compared to those without complications (*p* < 0.001). Furthermore, the proportion of patients in the lower TCBI-LN quartiles was markedly higher in the complication group (*P* < 0.001). Additionally, significant differences were observed between the two groups in terms of age, proportion of males, prevalence of malnutrition, and various inflammatory and nutritional markers (such as lymphocyte count, hemoglobin, albumin, and PNI), with all p-values < 0.05.

**Table 2:** Univariate analysis of clinical indicators and complications

Characteristics	Overall (*n* = 8,288)	Without Complications (*n* = 7,885)	With Complications (*n* = 403)	*P* value	Statistic
Sex, n (%)				<0.001	16.080
Male	5096 (61.5%)	4810 (61.0%)	286 (71.0%)		
Female	3192 (38.5%)	3075 (39.0%)	117 (29.0%)		
Age(year), mean ± sd	67.47 ± 13.81	67.25 ± 13.87	71.75 ± 11.77	<0.001	-7.412
Marital status, n (%)				0.401	0.704
Married	7977 (96.2%)	7586 (96.2%)	391 (97%)		
Single or divorced	311 (3.8%)	299 (3.8%)	12 (3%)		
Weight(kg), mean ± sd	63.97 ± 11.61	63.96 ± 11.60	64.16 ± 11.77	0.735	-0.339
Ethnicity, n (%)				0.741	0.110
Han Chinese	7984 (96.3%)	7597 (96.3%)	387 (96.0%)		
Others	304 (3.7%)	288 (3.7%)	16 (4.0%)		
Education Level, n (%)				<0.001	46.726
Bachelor’s degree or above	2622 (31.6%)	2512 (31.9%)	110 (27.3%)		
Junior high school or below	3702 (44.7%)	3541 (44.9%)	161 (40.0%)		
Senior high school	1011 (12.2%)	968 (12.3%)	43 (10.7%)		
Unknown	953 (11.5%)	864 (11.0%)	89 (22.1%)		
Payment Method, n (%)				0.045	8.026
Public funding (government)	931 (11.2%)	894 (11.3%)	37 (9.2%)		
Basic medical insurance	5405 (65.2%)	5120 (64.9%)	285 (70.7%)		
Out-of-pocket	1601 (19.3%)	1529 (19.4%)	72 (17.9%)		
Others	351 (4.2%)	342 (4.3%)	9 (2.2%)		
Hospital Department, n (%)				0.096	2.765
Medical department	5218 (63.0%)	4980 (63.2%)	238 (59.1%)		
Surgical department	3070 (37.0%)	2905 (36.8%)	165 (40.9%)		
Primary Diagnosis, n (%)				<0.001	102.597
Endocrine and Rheumatic Immune Diseases	278 (3.4%)	265 (3.4%)	13 (3.2%)		
Neurological and Psychiatric Diseases	1390 (16.8%)	1362 (17.3%)	28 (6.9%)		
Bone and Joint Diseases	532 (6.4%)	528 (6.7%)	4 (1.0%)		
Benign Digestive System Diseases	1406 (17%)	1350 (17.1%)	56 (13.9%)		
Benign genitourinary diseases	150 (1.8%)	135 (1.7%)	15 (3.7%)		
Benign Respiratory System Diseases	834 (10.1%)	761 (9.7%)	73 (18.1%)		
Cardiovascular Diseases	1217 (14.7%)	1166 (14.8%)	51 (12.7%)		
Malignant Tumors	2291 (27.6%)	2146 (27.2%)	145 (36.0%)		
Others	190 (2.3%)	172 (2.2%)	18 (4.5%)		
History of Diabetes, n (%)				0.001	10.539
Yes	1064 (12.8%)	991 (12.6%)	73 (18.1%)		
No	7224 (87.2%)	6894 (87.4%)	330 (81.9%)		
History of Hypertension, n (%)				0.006	7.470
Yes	2595 (31.3%)	2444 (31.0%)	151 (37.5%)		
No	5693 (68.7%)	5441 (69.0%)	252 (62.5%)		
History of Coronary Heart Disease, n (%)				0.566	0.329
Yes	1214 (14.6%)	1151 (14.6%)	63 (15.6%)		
No	7074 (85.4%)	6734 (85.4%)	340 (84.4%)		
History of Cerebral Infarction, n (%)				0.745	0.106
Yes	744 (9%)	706 (9.0%)	38 (9.4%)		
No	7544 (91%)	7179 (91.0%)	365 (90.6%)		
TCBI-LN, mean ± sd	7.08 ± 0.83	7.10 ± 0.83	6.83 ± 0.71	<0.001	7.195
Calf Circumference(cm), mean ± sd	33.13 ± 5.19	33.12 ± 5.26	33.29 ± 3.62	0.530	-0.627
Handgrip Strength(kg), mean ± sd	23.83 ± 13.87	23.93 ± 13.86	21.90 ± 14.08	0.004	2.876
BMI(kg/m^2^), mean ± sd	23.47 ± 3.58	23.47 ± 3.57	23.344 ± 3.81	0.512	0.656
NRS 2002, n (%)				<0.001	48.219
<3	5071 (61.2%)	4883 (61.9%)	188 (46.7%)		
3–5	2492 (30.1%)	2342 (29.7%)	150 (37.2%)		
≥ 5	725 (8.7%)	660 (8.4%)	65 (16.1%)		
Nutritional status (GLIM), n (%)				<0.001	17.792
Malnutrition	2397 (28.9%)	2243 (28.4%)	154 (38.2%)		
No malnutrition	5891 (71.1%)	5642 (71.6%)	249 (61.8%)		
WBC(×10⁹/L), mean ± sd	6.66 ± 2.88	6.65 ± 2.86	6.87 ± 3.25	0.193	-1.303
LYM(×10⁹/L), mean ± sd	1.90 ± 1.80	1.91 ± 1.82	1.58 ± 1.45	<0.001	4.472
Hb(g/L), mean ± sd	125.97 ± 19.72	126.27 ± 19.57	119.97 ± 21.68	<0.001	5.716
PLT(×10⁹/L), mean ± sd	208.22 ± 60.89	207.84 ± 61.07	215.80 ± 56.79	0.010	-2.564
PLR, mean ± sd	165.06 ± 172.33	163.80 ± 172.62	189.75 ± 164.80	0.003	-2.950
GLU(mg/dl), mean ± sd	108.39 ± 28.67	108.26 ± 28.67	110.97 ± 28.43	0.064	-1.856
TyG, mean ± sd	8.77 ± 0.76	8.78 ± 0.77	8.60 ± 0.63	<0.001	5.388
TBCI-Q, n (%)				<0.001	29.512
Quartile 1	2072 (25%)	1939 (24.6%)	133 (33%)		
Quartile 2	2072 (25%)	1966 (24.9%)	106 (26.3%)		
Quartile 3	2072 (25%)	1967 (24.9%)	105 (26.1%)		
Quartile 4	2072 (25%)	2013 (25.5%)	59 (14.6%)		
ALT(U/L), mean ± sd	24.75 ± 32.41	24.62 ± 31.92	27.253 ± 40.83	0.203	-1.275
TBIL(µmol/L), mean ± sd	14.12 ± 16.22	14.09 ± 16.10	14.78 ± 18.39	0.400	-0.841
BUN(mmol/L), mean ± sd	6.03 ± 3.51	6.00 ± 3.41	6.78 ± 5.07	0.002	-3.056
Cr(µmol/L), mean ± sd	74.95 ± 34.35	74.41 ± 32.96	85.39 ± 53.82	<0.001	-4.054
TG(mg/dl), mean ± sd	164.08 ± 176.70	166.23 ± 179.09	122.16 ± 112.86	<0.001	7.378
TC(mg/dl), mean ± sd	164.82 ± 59.70	165.27 ± 60.22	156.06 ± 47.66	0.003	3.020
ALB(g/L), mean ± sd	38.65 ± 5.11	38.75 ± 5.09	36.67 ± 5.04	<0.001	7.989
PNI, mean ± sd	48.13 ± 11.12	48.31 ± 11.16	44.56 ± 9.61	<0.001	7.591
TP(g/L), mean ± sd	65.65 ± 6.77	65.72 ± 6.74	64.30 ± 7.11	<0.001	4.111
Total length of hospital stay(days), mean ± sd	13.56 ± 5.92	13.29 ± 5.75	18.16 ± 7.08	<0.001	-13.577
ICU admission, n (%)				<0.001	17.961
Yes	411 (5.0%)	373 (4.7%)	38 (9.4%)		
No	7877 (95.0%)	7512 (95.3%)	365 (90.6%)		
Total healthcare costs(CNY), median (IQR)	19792.8 (10467.6, 45341.3)	19253.3 (10326.2, 43896.8)	37383.9 (16859.2, 76858.0)	<0.001	-8.861
In-hospital mortality, n (%)				<0.001	20.454
Yes	8260 (99.7%)	7864 (99.7%)	396 (98.3%)		
No	28 (0.3%)	21 (0.3%)	7 (1.7%)		

Based on the results of univariate analyses and the DAGs (Supplementary Fig. 1), we identified the necessary adjustment variables for multivariable logistic regression analysis to further confirm the independent association between TCBI-LN and complications. Multivariable logistic regression analysis further confirmed the independent association between TCBI-LN and complications (Table 3). This association remained significant after sequentially adjusting for potential confounding factors. When TCBI-LN was analyzed as a continuous variable, it demonstrated a consistently negative association with the risk of in-hospital complications across all models. In the unadjusted Model 1, each one-unit increase in TCBI-LN was associated with an approximately 36.3% reduction in the odds of complications (OR 0.637, 95% CI 0.558–0.729). This protective effect persisted after stepwise adjustment for covariates in Models 2–6, with ORs ranging from approximately 0.71 to 0.81 and 95% CIs all below 1. Additionally, we categorized TCBI-LN into quartiles and repeated the above modeling approach with trend tests. Compared to the lowest quartile of TCBI-LN, the highest quartile showed significantly reduced complication risks in Models 1–4, whereas the ORs for the second and third quartiles were close to 1, with most 95% CIs crossing 1, suggesting that modestly elevated TCBI-LN levels were not consistently associated with risk reduction. In Models 5–6, which included additional metabolic and nutritional indicators, the protective effect in the highest quartile was attenuated. The overall trend test was no longer significant in the more complex models, indicating that some gradient effects may be explained by other covariates. All VIF values in the models presented in Table 3 were < 2.0, indicating no significant multicollinearity issues.

**Table 3:** Predictive value of TCBI-LN for clinical outcomes

Variables	Model1	Model2	Model3	Model4	Model5	Model6
	OR(95% CI)	OR(95% CI)	OR(95% CI)	OR(95% CI)	OR (95% CI)	OR (95% CI)
TCBI-LN	0.637 (0.558–0.729)***	0.687 (0.597–0.790)***	0.737 (0.633–0.857)***	0.706 (0.598–0.833)***	0.775 (0.608–0.987)*	0.813 (0.697–0.948)**
TCBI-LN quartiles						
Quartile 1 (*n* = 2072)	1	1	1	1	1	1
Quartile 2 (*n* = 2072)	0.780 (0.600-1.014)	0.785 (0.603–1.023)	0.774 (0.592–1.012)	0.760 (0.577-1.000)	0.879 (0.657–1.177)	0.865 (0.658–1.137)
Quartile 3 (*n* = 2072)	0.772 (0.593–1.004)	0.822 (0.630–1.071)	0.842 (0.642–1.105)	0.842 (0.617-1.100)	1.022 (0.734–1.422)	1.001 (0.756–1.325)
Quartile 4 (*n* = 2072)	0.417 (0.304–0.570)***	0.505 (0.367–0.696)***	0.593 (0.428–0.821)***	0.564 (0.398–0.799)***	0.797 (0.500–1.270)	0.715 (0.512-1.000)
P for trend	<0.001	<0.001	0.005	0.004	0.647	0.153

Model 1: Unadjusted

Model 2: Adjusted for sex, age, marital status, education level, ethnicity

Model 3: Adjusted for sex, age, marital status, education level, ethnicity, disease type, hospital department and the presence of hypertension, diabetes, coronary heart disease, or cerebral infarction

Model 4: Adjusted for all variables in Model 3 plus the CC, HGS, and the presence of malnutrition

Model 5：Adjusted for all variables in Model 3 plus the TyG , PLR, and PNI

Model 6：Adjusted for all variables in Model 3 plus disease severity scores

*P < 0.05; **P < 0.01; ***P < 0.001

**Fig. 5:**
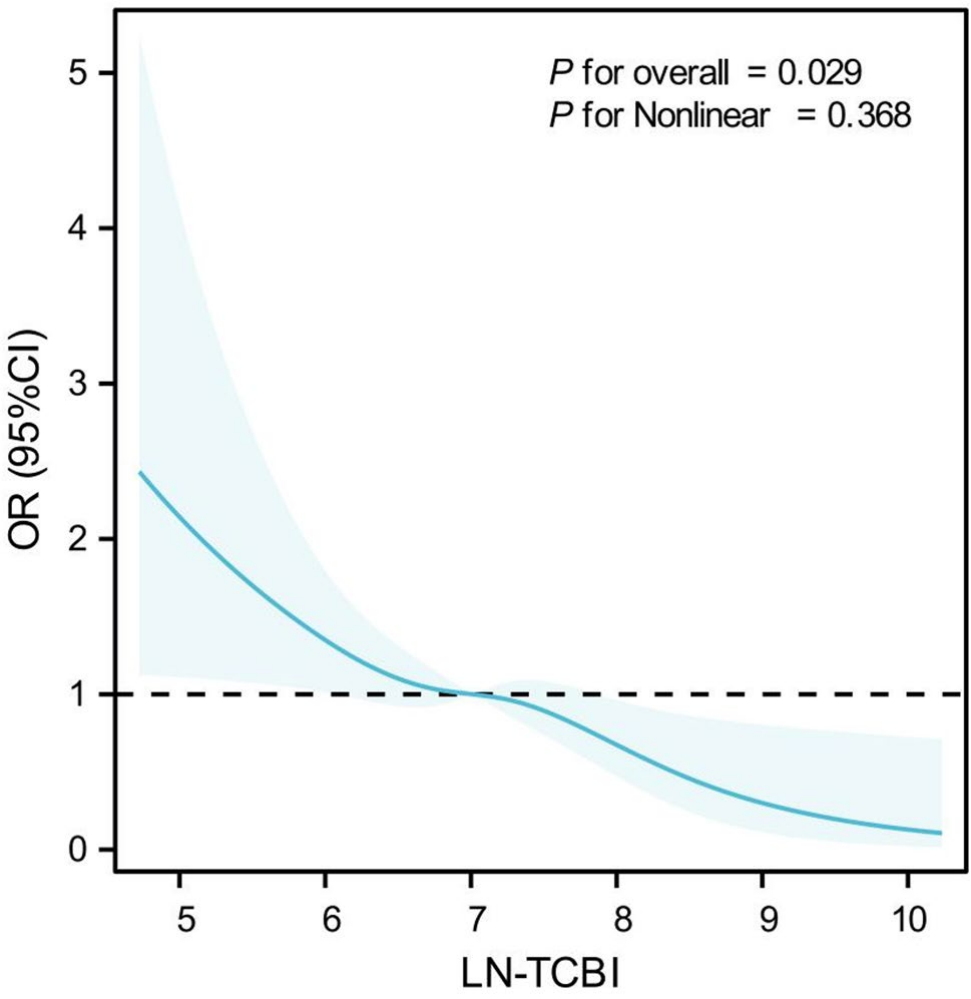
Restricted cubic spline plot

As shown in Fig. 5, the RCS analysis indicated an overall linear and monotonically decreasing relationship between TCBI-LN and the risk of in‑hospital complications, with no significant nonlinear pattern observed. The overall association test was statistically significant (P for overall = 0.029), suggesting that across the entire sample, TCBI‑LN as a continuous variable is significantly associated with complication risk. The nonlinearity test was not significant (P for nonlinear = 0.368), indicating that within the modeled range, the relationship between TCBI‑LN and the log‑odds of complications can be reasonably approximated by a linear function, without evident thresholds or inflection points. Together, the RCS curve supports a linear dose‑response pattern—higher TCBI‑LN is associated with lower in‑hospital complication risk—and underscores the stable risk‑stratification capacity of TCBI‑LN as a continuous measure.

### Association between TCBI-LN and specific types of in-hospital complications

We further categorized complications into infection-related, non-infection-related, and both types combined for analysis (Table 4). The results showed a decreasing trend in the incidence of all complication types with increasing TCBI-LN quartiles (from Q1 to Q4), and the differences were statistically significant (*p* < 0.001). The Q4 group had the lowest incidence rates across all complication categories.

**Table 4:** Association between TCBI-LN quartiles and specific types of in-hospital complications

TCBI-LN	No complications (*n* = 7885)	Infection-related only (*n* = 252)	Non-infection-related only (*n* = 75)	Both (*n* = 76)	*P* value	Statistic
TCBI-LN, mean ± sd	7.10 ± 0.83	6.80 ± 0.74	6.85 ± 0.68	6.85 ± 0.83	<0.001	23.941
					<0.001	36.920
Quartile 1(*n* = 2072)	1938(93.5%)	87(4.2%)	28(1.4%)	19(0.9%)		
Quartile 2(*n* = 2072)	1966(94.9%)	61(2.9%)	19(0.9%)	26(1.3%)		
Quartile 3(*n* = 2072)	1967(94.9%)	65(3.1%)	18(0.9%)	22(1.1%)		
Quartile 4(*n* = 2072)	2014(97.2%)	39(1.9%)	10(0.5%)	9(0.4%)		

### Stratified analysis

In this study, the cut-off value for risk stratification was determined based on the maximum Youden index. Specifically, the AUC was calculated as 0.670 (95% CI 0.650–0.700) using DeLong’s method, and the point corresponding to the maximum Youden index was selected as the first cut-off value, which was 953.6. To achieve further stratification, we repeated the above procedure among patients with TCBI ≥ 953.6. The AUC for this subgroup was 0.691 (95% CI 0.654–0.729), and the point corresponding to the maximum Youden index was selected as the second cut-off value, which was 1525.0. Based on these sequential cut-offs, the study population was stratified into low-risk, intermediate-risk, and high-risk groups (Table 5). Calibration analyses were performed for all the above results (Supplementary Figs. 2–3), and internal validation was conducted (Supplementary Tables 1–2), demonstrating robust findings. Stratified analysis revealed that the incidence of complications was significantly higher in the high-risk group compared to the intermediate- and low-risk groups (6.3%, 5.1%, and 2.9%, respectively).

**Table 5:** Complication rates across risk strata defined by TCBI cut-off values

	High-risk group	Intermediate-risk group	Low-risk group	Total	*P*	Statistic
TCBI range	<953.6	953.6 to < 1525.0	≥ 1525.0		<0.001	37.901
With in-hospital complications	215(6.3%)	110(5.1%)	78(2.9%)	403		
Without in-hospital complications	3214(93.7%)	2043(94.9%)	2628(97.1%)	7885		
Total	3429	2153	2706			

### Adjusted subgroup analysis and interaction tests

**Fig. 6:**
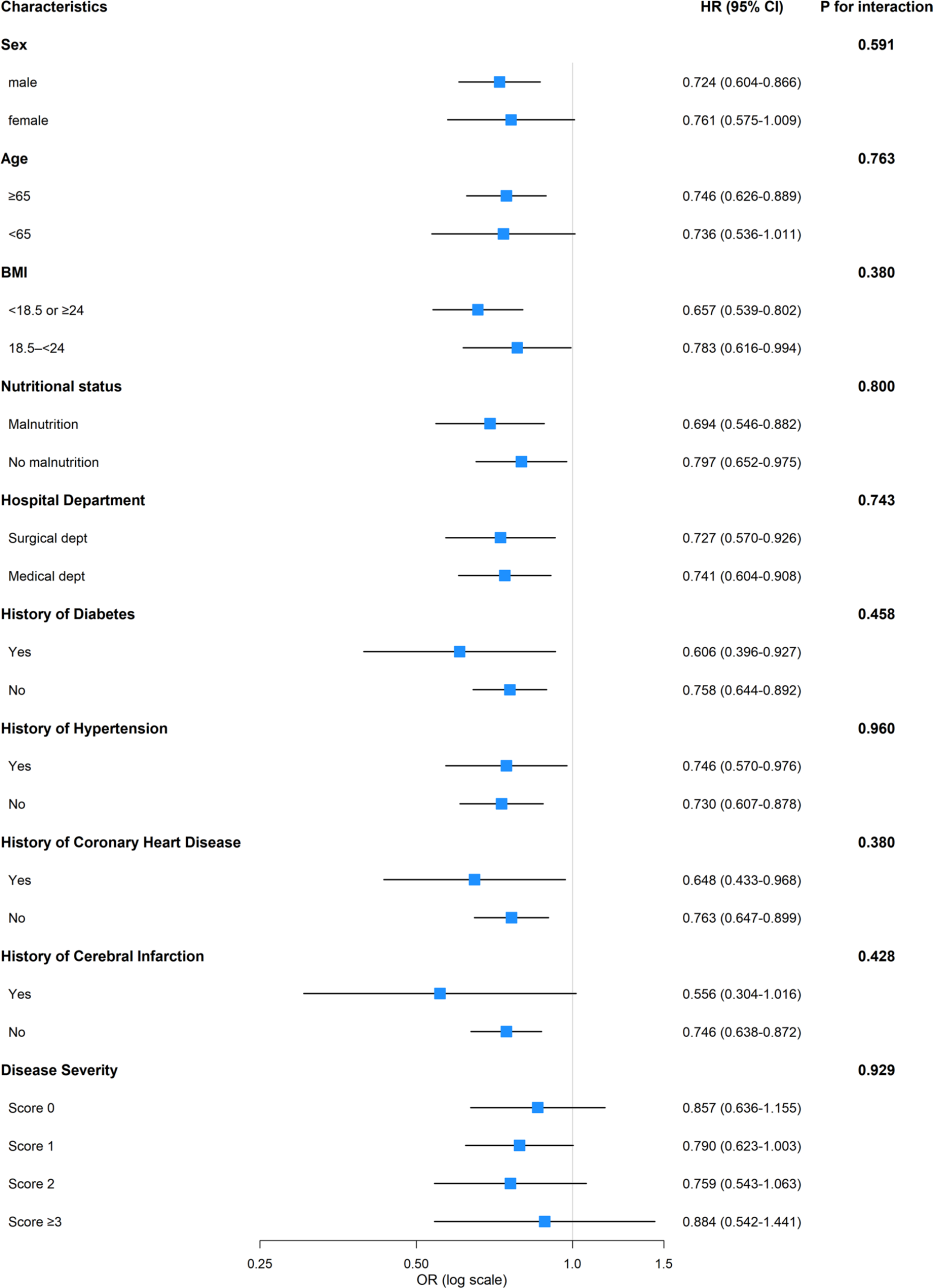
Forest plot for interaction analysis (Adjusted for variables in Model 3)

To evaluate the robustness of the association between TCBI-LN and complication occurrence, we performed stratified logistic regression subgroup analyses and interaction tests after adjusting for the covariates included in Model 3 (Fig. 6). In pre-specified subgroup analyses, the negative association between higher TCBI-LN and in-hospital complications was generally consistent across clinically relevant strata. The protective association of TCBI-LN was observed in both males and females, with no evidence of interaction by sex. Similar risk reductions were demonstrated in patients aged ≥ 65 years and those aged < 65 years. Across different BMI categories, nutritional status, hospital departments, and histories of diabetes, hypertension, coronary heart disease, and cerebral infarction, higher TCBI-LN remained associated with lower complication risk, with none of the interaction terms reaching statistical significance. When stratified by disease severity score, the direction of the association between TCBI-LN and complications was consistently protective across all categories; however, CI were wide and mostly crossed 1.0, and no significant interaction was detected. Collectively, these findings suggest that the beneficial association of higher TCBI-LN with reduced in-hospital complications is broadly robust, with no substantial differences observed across the examined patient subgroups.

### Sensitivity analysis

To evaluate potential clustering effects at the hospital level, we performed sensitivity analyses. As shown in Supplementary Table 3, we first employed multilevel mixed-effects logistic regression models with hospital as a random intercept, and the results were largely consistent with the main analysis, with an intraclass correlation coefficient (ICC) < 0.01%, indicating minimal between-hospital variation. Second, analyses using GEE with cluster-robust sandwich standard errors also confirmed the association. The consistency of results across the three models suggests that hospital-level clustering effects have a negligible impact on the primary findings. Additionally, we conducted sensitivity analyses using binary logistic regression stratified by LOS (Supplementary Table 4) and by whether patients experienced ICU admission during hospitalization (Supplementary Table 5). The results consistently demonstrated the robustness of the association between TCBI-LN and complications (*P*<0.05).

### Comparative predictive performance of TCBI vs. components and existing indices

In the comparison of different predictive models (Table 6), Model A, which included only traditional clinical covariates, yielded an AUC of 0.683 and served as the baseline model. Model B, which added the three individual components of TCBI (TG, TC, and BW) to the baseline, achieved an AUC of 0.693, showing a statistically significant improvement compared to Model A. Additionally, AIC and BIC values decreased, with an NRI of 0.141 and an IDI of 0.002, suggesting modest enhancements in both model discrimination and reclassification capability. Model C, which replaced the individual components with the composite TCBI-LN index, demonstrated an AUC of 0.694, with slightly higher NRI and IDI values than Model B, along with further reductions in AIC and BIC. This indicates that the composite TCBI-LN provides more efficient predictive information than including TG, TC, and BW separately. Model D, which added the commonly used nutritional/metabolic indices PNI and TyG to the clinical model, achieved an AUC of 0.701 and obtained the highest NRI and IDI among this group, suggesting that PNI and TyG contribute more substantially to model performance improvement. When TCBI-LN, PNI, and TyG were all included in the comprehensive Model E, the AUC reached the highest value of 0.703, with an AIC of 3042.9—the lowest among all models—while maintaining relatively high NRI and IDI values. These findings demonstrate that TCBI-LN provides independent incremental predictive value beyond traditional clinical factors and existing nutritional indices, and that the combination of TCBI-LN with PNI and TyG yields the optimal overall predictive performance.

**Table 6:** Comparison of predictive performance across different models

Model	AUC (95% CI)	DeLong *P* vs. Model A	LRT *P* vs. Model A	AIC	BIC	NRI (95% CI)	IDI (95% CI)
Model A: Clinical covariates only*	0.683 (0.659–0.707)	—	—	3072.2	3191.5	—	—
Model B: Model A + TG + TC + BW	0.693 (0.668–0.717)	0.009	< 0.001	3061.5	3201.9	0.141 (0.044–0.240)	0.002 (0.001–0.003)
Model C: Model A + TCBI-LN	0.694 (0.670–0.719)	0.017	< 0.001	3054.1	3180.5	0.171 (0.070–0.273)	0.003 (0.001–0.004)
Model D: Model A + PNI + TyG	0.701 (0.678–0.726)	0.001	< 0.001	3046.8	3180.2	0.243 (0.144–0.349)	0.004 (0.002–0.006)
Model E: Model A + TCBI-LN + PNI + TyG	0.703 (0.678–0.727)	< 0.001	< 0.001	3042.9	3183.3	0.210 (0.110–0.312)	0.005 (0.003–0.008)

*Sex, age, disease type, hospital department and the presence of hypertension, diabetes, coronary heart disease, or cerebral infarction

### Association between TCBI-LN and health economics outcomes

**Fig. 7:**
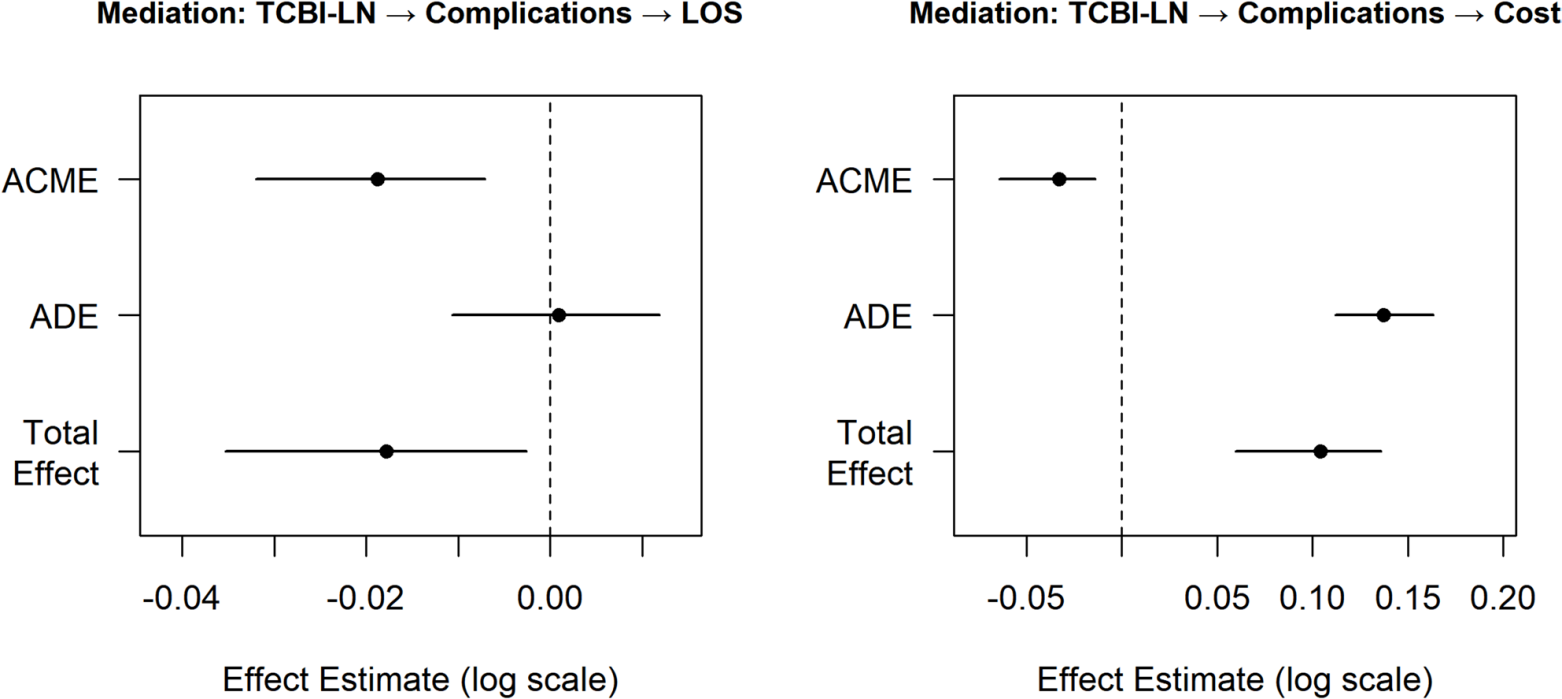
Mediation pathway diagram: The mediating role of complications in the relationship between TCBI-LN and LOS and HC

Mediation analysis was performed to explore the relationship between TCBI-LN and both LOS and HC (Table 7; Fig. 7). The total effect of TCBI-LN on LOS was significant, indicating that higher TCBI was associated with shorter LOS. The average causal mediation effect (ACME) through complications was − 0.019, while the average direct effect (ADE) was negligible and non-significant. The proportion mediated was 105.3%, suggesting that complications fully mediated the relationship between TCBI-LN and LOS—the beneficial effect of higher TCBI on hospitalization duration was entirely explained by its protective association with in-hospital complications. For HC, a discordant mediation pattern was observed. Although the ACME through the complication pathway was protective (β = −0.033, *P* < 0.001), the direct effect of TCBI-LN on HC was positive and larger in magnitude (β = 0.137, *P* < 0.001), potentially reflecting higher baseline treatment costs among patients with greater body weight and lipid levels. The net total effect was positive (β = 0.1043, *P* < 0.001), and the negative proportion mediated (− 31.6%) indicated that the complication pathway acted as a suppressor, attenuating what would otherwise be a larger direct increase in HC.

**Table 7:** Mediation analysis results for TCBI-LN, complications, LOS, and HC

Effect	Estimate_95CI	P_value	Outcome
ACME	-0.019 (-0.032–-0.007)	< 0.001	log (LOS)
ADE	0.001 (-0.011–0.012)	0.898	log (LOS)
Total Effect	-0.018 (-0.035–-0.003)	0.032	log (LOS)
Prop. Mediated	1.053 (0.515–3.166)	0.032	log (LOS)
ACME	-0.033 (-0.064–-0.014)	< 0.001	log (HC)
ADE	0.137 (0.112–0.163)	< 0.001	log (HC)
Total Effect	0.104 (0.060–0.136)	< 0.001	log (HC)
Prop. Mediated	-0.316 (-0.987–-0.111)	< 0.001	log (HC)

*ACME* Average causal mediation effect (indirect effect), *ADE* Average direct effect, *Prop*. *Mediated* Proportion mediated

## Discussion

This large multicenter prospective cohort study is the first to systematically evaluate the association between admission TCBI and in-hospital complications in a Chinese hospitalized population. TCBI-LN approximated a normal distribution, with lower levels independently associated with higher complication risk after comprehensive adjustment for demographics, clinical parameters, and nutritional indices. This association demonstrated generalizability across diverse patient subgroups. Furthermore, TCBI-LN provided incremental predictive value beyond traditional covariates and established indices (PNI, TyG), with the comprehensive model achieving the highest AUC, greatest reclassification improvement, and lowest AIC. Mediation analysis showed that complications fully mediated the TCBI-LN-LOS association and partially offset the higher costs linked to elevated TCBI-LN, underscoring its potential as a risk stratification tool.

TCBI was initially proposed by Doi et al. [20] in patients with coronary artery disease as a simple index incorporating TG, TC, and BW to reflect the body’s lipid reserves and nutritional status. In that and subsequent cardiovascular cohort studies, a lower TCBI was associated with a higher risk of all-cause mortality and major adverse cardiovascular events, interpreted as an adverse prognostic signal reflecting “undernutrition and decreased lipid reserves” [21]. In contrast to these studies, our research included a mixed medical-surgical inpatient population and focused on in-hospital composite complications rather than long-term mortality or cardiovascular events. Our results indicate that, even within the real-world setting of general wards, a low TCBI-LN similarly signifies a higher short-term risk of adverse events. This finding extends the potential application of TCBI beyond the context of cardiovascular diseases.

Our study found that TCBI-LN was inversely associated with the risk of in-hospital complications, meaning a higher TCBI-LN predicted a lower incidence of complications. This protective association with complications, however, does not uniformly translate to lower ICU admission rates, as the latter is also strongly influenced by disease-specific monitoring practices—exemplified by the disproportionately high ICU admissions among neurological and benign genitourinary patients in the highest TCBI-LN quartile despite their lower complication burden. This aligns with the physiological significance reflected by its component variables. TCBI is calculated as the product of BW, TG, and TC; thus, a higher TCBI-LN generally indicates relatively sufficient energy reserves and active lipid metabolic turnover. Firstly, adequate BW and nutritional status form the foundation for maintaining immune function, tissue repair, and stress response capacity. Although extremely high TG and TC levels are cardiovascular risk factors [21], moderate lipid levels in the acute illness setting may reflect preserved hepatic synthetic function and energy substrate availability. In our study, patients with complications actually had significantly lower TG and TC levels than those without. A possible explanation is that our primary endpoint focused on short-term, in-hospital composite complications, which are more susceptible to influences from nutritional status, energy reserves, and acute inflammatory responses, whereas atherosclerosis-related events typically represent medium- to long-term outcomes. Furthermore, our models adjusted for multiple covariates related to cardiovascular risk and inflammation, which may have partially attenuated the direct impact of “hyperlipidemia” on short-term complications. This observation is consistent with findings from other studies suggesting that very low lipid levels may be associated with severe inflammatory responses, hepatic dysfunction, and poor prognosis [22–24]. Therefore, TCBI-LN may represent a physiological state of relatively adequate “nutritional-metabolic reserve,” enabling patients to better withstand the stress of acute illness during hospitalization.

In this study, TCBI-LN was positively correlated with nutritional indicators such as albumin, PNI, and CC. Furthermore, the lowest TCBI-LN quartile (Q1) had the highest proportions of patients diagnosed with malnutrition by GLIM criteria and those with NRS 2002 scores ≥ 3. This aligns with previous studies confirming nutritional status as a core determinant of complications [8, 19]. Additionally, TCBI-LN showed positive correlations with metabolic markers like TG, TC, and the TyG index, but negative correlations with age and inflammatory markers such as PLR. This pattern suggests that TCBI-LN integrates dual information on both nutrition and metabolism.

Compared to traditional single indicators, TCBI-LN demonstrated unique predictive advantages. For example, although BMI is a commonly used nutritional assessment parameter, it showed no significant difference between patients with and without complications in our univariate analysis (*p* = 0.512), whereas TCBI-LN did. This indicates that BMI alone may fail to capture information about metabolic composition, whereas TCBI, by incorporating lipid parameters, provides a more nuanced profile. Similarly, the TyG index, which has garnered considerable research attention recently [12], was also inversely associated with complication risk in our study. When included as an adjustment factor in Model 5, TCBI-LN remained independently significant, suggesting that TCBI-LN may convey information beyond insulin resistance that is relevant to complication risk, encompassing other metabolic and nutritional dimensions. Moreover, the negative correlation between TCBI-LN and the inflammatory marker PLR supports the concept of a close link between nutritional-metabolic status and systemic inflammation [25]. Adequate nutritional reserves may help mitigate excessive inflammatory responses, while low-grade inflammation can exacerbate metabolic dysregulation and malnutrition, creating a vicious cycle [26]. TCBI-LN may indirectly reflect this state of physiological balance. Our mediation analysis further supported the above hypotheses from a causal pathway perspective. When LOS was the outcome, the total effect between TCBI-LN and LOS was almost entirely mediated through the pathway of in-hospital complications, with the direct effect no longer significant. This suggests that nutritional-metabolic status primarily shortens hospitalization duration by reducing complications, consistent with previous findings that “complications are a key determinant of prolonged LOS” [2–5]. When hospitalization costs were the outcome, a discordant mediation pattern emerged: although patients with higher TCBI-LN incurred higher baseline treatment costs due to increased body weight and metabolic burden, this cost increase was partially offset by reduced complications, reflecting the potential value of nutritional optimization in conserving complication-related resource consumption.

Furthermore, this study suggests a complementary relationship between TCBI and traditional nutritional assessment tools. The PNI, primarily based on albumin and lymphocyte count, emphasizes protein nutrition and immune function [14]. The NRS-2002 and GLIM criteria, incorporating weight change, dietary intake, and disease burden, focus more on nutritional intake and body composition changes [27]. In contrast, TCBI highlights lipid reserves and body mass dimensions and can be quickly derived from routine lipid profiles. In our multivariate models, TCBI-LN remained independently associated with complications even after including these established nutritional indicators. This finding supports the potential utility of TCBI as a valuable supplement to, rather than a simple replacement for, existing nutritional screening tools in real-world mixed medical-surgical inpatient populations.

Our study confirms that TCBI-LN is not only a significant predictor but also a practical biomarker derivable from routine tests, meeting the clinical need outlined earlier. Despite providing compelling evidence, several limitations of this study must be acknowledged. First, the TCBI index itself has inherent limitations. It cannot distinguish between different body composition components (e.g., fat versus muscle mass). A high TCBI value may result from obesity with hyperlipidemia, or alternatively, from normal muscle mass with moderate lipid levels, with the former potentially associated with increased risk of specific complications in certain clinical contexts [8]. Although we adjusted for BMI and CC, more precise body composition measurements were not available. Second, this study utilized a single TCBI measurement at admission as the exposure variable, which failed to capture dynamic changes in TCBI or related metabolic-nutritional indicators during hospitalization. Third, as the cohort predominantly consisted of hospitalized patients from China, there may be patient and hospital heterogeneity, and caution is warranted when generalizing these findings to other ethnicities, populations, or outpatient settings. Fourth, although the use of a composite complication endpoint aligns with the holistic focus of clinical practice, different types of complications (e.g., infectious, cardiovascular, hemorrhagic, and surgery-related complications) and varying disease severity may involve distinct pathophysiological mechanisms. The predictive performance of TCBI for specific complication categories requires further validation through subtype analyses. Fifth, detailed documentation of specific Clavien-Dindo grades for complications was not available in this study. Although supplementary analyses of specific complication categories and severity grades demonstrated consistent associations, residual heterogeneity may still exist. Finally, this study only included patients with a LOS of 7–30 days. While this criterion allows for a more accurate characterization of cyclical changes in patient nutritional status and sensitivity analyses yielded consistent results, we may have excluded patients with extremely mild (short-stay) or highly complex (long-stay) conditions, which could potentially affect the generalizability of our conclusions.

## Conclusion

This real-world study demonstrates that TCBI-LN is a composite indicator capable of simultaneously reflecting the nutritional and metabolic status of hospitalized patients. An elevated TCBI-LN is independently associated with a reduced risk of in-hospital complications. This association remains consistent across various patient subgroups and exhibits a linear dose-response relationship. Although the index does not differentiate body composition, its simplicity and cost-effectiveness give it potential as an early warning tool for complications in hospitalized patients. Further mechanistic and interventional studies are warranted to validate these findings.

## Supplementary Information

Supplementary Material 1.

## Data Availability

No datasets were generated or analysed during the current study.
