# Supplementary material for: Lower triglyceride-cholesterol-body weight index is independently associated with increased in-hospital complication risk: a large multicenter real-world study
Source: Lipids Health Dis. 2026 Apr 9;25:132. doi: 10.1186/s12944-026-02947-w (PMC13188760; doi:10.1186/s12944-026-02947-w)
Supplement: Supplementary file 1 — Supplementary Material 1. [file 12944_2026_2947_MOESM1_ESM.docx]

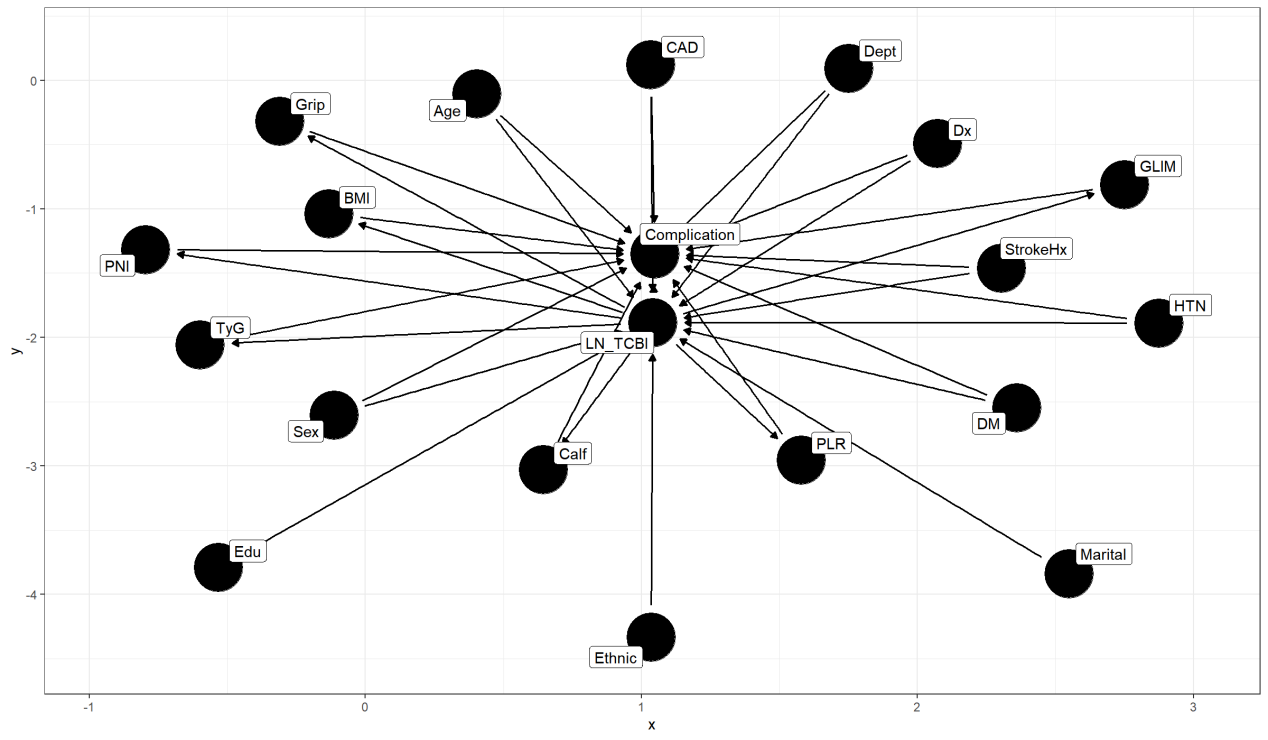


Supplementary Figure 1. Directed Acyclic Graphs.


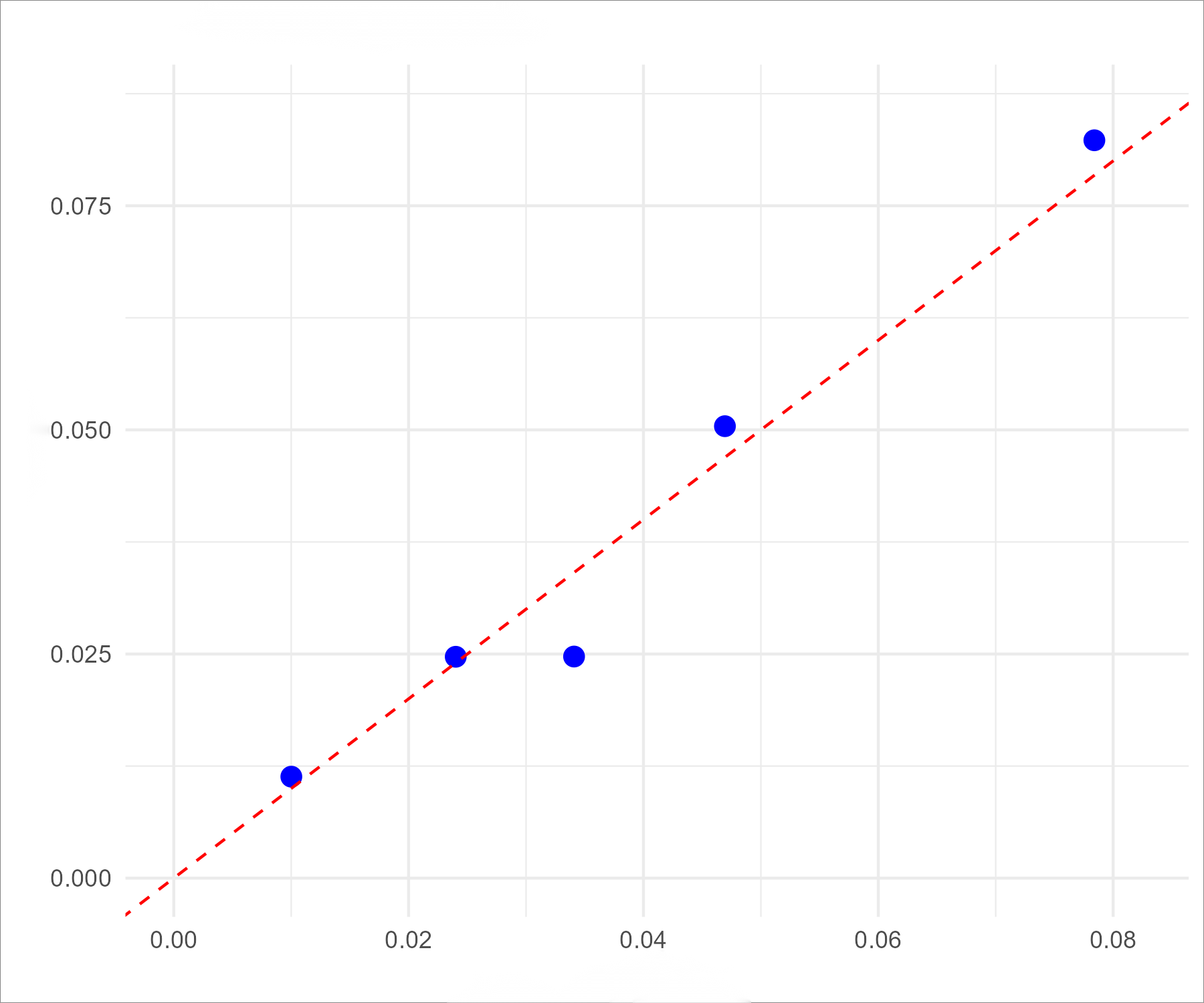


Supplementary Figure 2. Calibration curve at the first TCBI cutoff (953.6)

Note: The vertical axis represents the observed incidence of in‑hospital complications, and the horizontal axis represents the predicted incidence of in‑hospital complications.


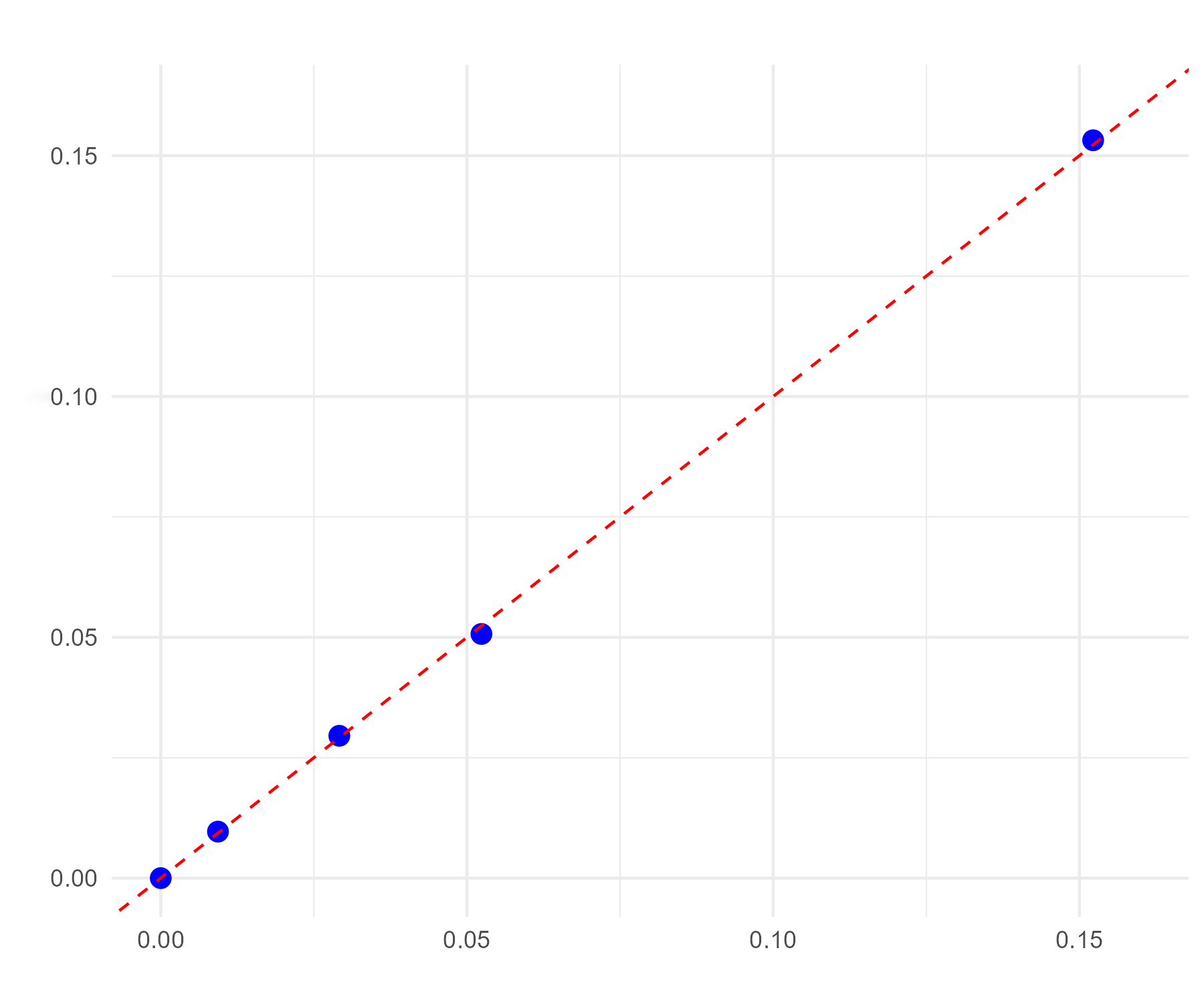


Supplementary Figure 3. Calibration curve at the second TCBI cutoff (1525.0)

Note: The vertical axis represents the observed incidence of in‑hospital complications, and the horizontal axis represents the predicted incidence of in‑hospital complications.

Supplementary Table 1. Area under the curve (AUC) and internal validation results at the first TCBI cutoff (953.6)

| **Original AUC (95% CI)** | **Bootstrap Mean** | **Bootstrap 95% CI** | **Optimism** | **Corrected AUC** |
| --- | --- | --- | --- | --- |
| 0.670 (0.650-0.700) | 0.68 | 0.650-0.700 | 0.006 | 0.670 |

Supplementary Table 2. Area under the curve (AUC) and internal validation results at the second TCBI cutoff (1525.0)

| **Original AUC (95% CI)** | **Bootstrap Mean** | **Bootstrap 95% CI** | **Optimism** | **Corrected AUC** |
| --- | --- | --- | --- | --- |
| 0.691 (0.654-0.729) | 0.7 | 0.661-0.735 | 0.009 | 0.683 |

Supplementary Table 3. Stratified analysis by hospital level (adjusted for variables in Model 3)

| **Model** | **Beta** | **SE** | **OR(95% CI)** | **P value** |
| --- | --- | --- | --- | --- |
| Base Logistic Regression | -0.5248 | 0.1728 | 0.592(0.422-0.830) | ＜0.001 |
| Mixed-effects (random intercept) | -0.5248 | 0.1728 | 0.592(0.422-0.830) | ＜0.001 |
| GEE (exchangeable + robust SE) | -0.5251 | 0.1466 | 0.592(0.444-0.788) | ＜0.001 |

Supplementary Table 4. Stratified analysis by categories of length of stay (LOS) (adjusted for variables in Model 3)

| **LOS Categories** | **OR(95% CI)** | **P value** |
| --- | --- | --- |
| 7-14 days (n=5,380) | 0.710(0.569-0.887) | 0.002 |
| 15-30 days (n=2,908) | 0.639(0.541-0.755) | ＜0.001 |

Supplementary Table 5. Sensitivity analysis according to ICU admission status (adjusted for variables in Model 3)

| **ICU admission status** | **OR(95% CI)** | **P value** |
| --- | --- | --- |
| Excluding ICU patients | 0.636(0.550-0.735) | <0.001 |
| ICU patients only | 0.444(0.313-0.631) | <0.001 |
